# At-Homeness: Rethinking Personhood-in-Community Through the Lens of Social Identity

**DOI:** 10.1093/geroni/igab046.1534

**Published:** 2021-12-17

**Authors:** Daniel R Y Gan, Graham Rowles, Habib Chaudhury

**Affiliations:** 1 Simon Fraser University, Vancouver, British Columbia, Canada; 2 University of Kentucky, Lexington, Kentucky, United States

## Abstract

Since Chaudhury’s seminal work (2008), spatial ethnographies of the everyday lives of people living with dementia(PLWD) have proliferated. From an experiential perspective, geographies of home (Blunt & Varley, 2004) and geographies of dementia may overlap significantly. We conducted a meta-ethnographic synthesis of n=28 articles to identify points of convergence and divergence in these literatures using comparative thematic analysis with NVivo 12. Whereas geographies of home highlight at-homeness (e.g., ontological safety and daily activities), geographies of dementia underscore communal and civic participation (e.g., social relationships). These themes converge around “social identity” which may be an important construct that helps PLWD feel at home. The quality of life of PLWD in the community may be influenced by prior (and present) experiences of at-homeness. These become more pertinent as older adults shelter in place. We discuss the implications of these findings in relation to relational models of personhood-in-community (Swinton, 2020) and community gerontology.

